# STB-HO, a novel mica fine particle, inhibits the teratoma-forming ability of human embryonic stem cells after *in vivo* transplantation

**DOI:** 10.18632/oncotarget.6472

**Published:** 2015-12-04

**Authors:** Soon Won Choi, Tae-Hoon Shin, Md. Hafiz Uddin, Ji-Hee Shin, Tae-Wook Kang, Byung-Chul Lee, Hyung-Sik Kim, Yoojin Seo, Sulaiman Shams, Yeon-Kwon Jung, Kyung-Sun Kang

**Affiliations:** ^1^ Adult Stem Cell Research Center, College of Veterinary Medicine, Seoul National University, Seoul, Republic of Korea; ^2^ Research Institute for Veterinary Science, College of Veterinary Medicine, Seoul National University, Seoul, Republic of Korea; ^3^ Institute for Stem Cell and Regenerative Medicine in Kangstem Biotech, Biomedical Science Building, College of Veterinary Medicine, Seoul National University, Seoul, Republic of Korea; ^4^ Stem Cells Regenerative Medicine Lab, Department of Biochemistry, Abdul Wali Khan University, Khyber Pakhtunkhwa, Pakistan; ^5^ Seobong BioBesstech Co., Ltd., Yeoksam-dong, Kangnam-gu, Seoul, Republic of Korea

**Keywords:** STB-HO, mica fine particle, teratoma formation, pluripotent stem cell, apoptosis

## Abstract

Although pluripotent stem cell (PSC) therapy has advantages for clinical applications because of the self-renewal and multi-lineage differentiation abilities of PSCs, it also has disadvantages in terms of the potential for PSCs to undergo malignant transformation or unexpected differentiation. The prevention of teratoma formation is the largest hurdle of all. Despite intensive studies that have investigated ways to block teratomas, such methods have yet to be further developed for clinical use. Here, a new approach has focused on exerting anti-tumorigenic effects using a novel mica fine particle (MFP) designated STB-HO. Treatment with STB-HO regulated pluripotency- and apoptosis-related genes in differentiating human embryonic stem (hES) cells, while there is no effects in undifferentiated hES cells. In particular, STB-HO blocked the anti-apoptotic gene BIRC5 and activated p53, p21 and the pro-apoptotic proteins Bim, Puma and p-Bad during early spontaneous differentiation. Moreover, STB-HO-pretreated differentiating hES cells did not give rise to teratomas following *in vivo* stem cell transplantation. Our *in vitro* and *in vivo* results suggest a method for teratoma prevention in the context of PSC-derived cell transplantation. This novel MFP could break through the limitations of PSC therapy.

## INTRODUCTION

Stem cell therapy has emerged as a therapeutic strategy with great potential in the last few years. The ultimate goals of stem cell therapy are tissue replacement and regeneration in degenerative diseases [1-3]. Stem cells, particularly human pluripotent stem cell (PSC), self-renew indefinitely and can differentiate into all cell types of the human body. With this excellent potential, they can be induced to differentiate into tissue-specific functional cells under proper cell culture conditions. Recently, such cell-specific differentiation protocols have improved and have been developed to obtain therapeutically relevant cell types from human PSC sources [4]. Most importantly, the generation of lineage-specific cells using PSCs has been investigated as a promising approach to understand and cure degenerative disorders such as Parkinson's disease, which involves dopamine neurons [5, 6], and Huntington's disease, which involves striatal neurons [7], as well as non-neural cell types in the liver [8] and pancreatic islets [9]. Despite excellent cell sources for the replacement of degenerative tissues, the clinical application of PSC therapies is strongly limited due to the potential for PSCs to form teratomas [10, 11]. Unless this risk is eliminated before transplantation, the teratoma issue is a central safety hurdle for the application of PSC therapies in regenerative medicine.

For PSC therapy, undifferentiated cells are differentiated *in vitro* toward certain tissue-specific cell types. During this differentiation process, PSCs can remain in an undifferentiated state in a mixture with their differentiated progeny and spontaneously give rise to teratomas after *in vivo* transplantation [12]. Therefore, numerous techniques have been attempted to prevent teratoma formation, and reduced incidence rates have been achieved, for example, via genetic modification of the herpes simplex virus thymidine kinase gene [13] and sorting of undifferentiated cells using SOX1 or SSEA-5 [14] as well as long-term culture during differentiation [15]. However, those techniques are not feasible solutions for clinical use. Alternative approaches have also been employed, such as the selective elimination of residual undifferentiated PSCs via transient treatment with monoclonal antibody 84 [16] as well as small molecules to target the remaining undifferentiated PSCs [17, 18], as recently reported.

Postulating that undifferentiated cells can be selectively eliminated *in vitro* before cell transplantation, the underlying mechanism must be understood for employment in PSC therapy. According to seminal studies, undifferentiated PSCs are very sensitive to DNA damage and are therefore fragile, undergoing programmed cell death (apoptosis). The promotion of apoptosis is caused not only by the tumor suppressor protein p53 but also by mitochondrial priming with the Bcl-2 protein family, which consists of initiators (BH3-only proteins), guardians (the pro-survival proteins) and effectors (the pro-apoptotic proteins) [9, 19]. Importantly, mitochondrial priming that exceeds the apoptotic threshold differs between PSCs and differentiated cells. A reliable study reported that BH3-only proteins were highly expressed in PSCs and were then gradually down-regulated upon differentiation [20].

Exploring new approaches to induce the selective elimination of undifferentiated cells, we tested a mica fine particle (MFP). In many previous studies, mica was studied in the context of immune regulation and demonstrated immune enhancing effects by activating macrophages [21, 22]. Another recent study investigated global cell responses of macrophages to a newly developed MFP using a microarray approach [23]. This microarray analysis reported huge changes in gene expression after treatment with MFP. Interestingly, MFP treatment markedly down-regulated genes related to the cell cycle (Mybl2, Cdc20, Rrm2, Ccne2), cell proliferation (Ki67), DNA replication (Mcm5, Mcm6) and DNA repair (Rad54l), whereas apoptosis-related genes (Gadd45a, Gadd153, Cd274) were increased by more than 8-fold. Although this study utilized the murine leukemic monocyte macrophage line *RAW 264.7*, the results implied that MFP could be involved in the selective elimination of undifferentiated cells, which are characterized by mitochondrial priming.

STB-HO is one of the MFP and recently developed to have the anti-tumor and the immune-stimulatory effects. A cancer study using STB-HO has reported chemo-preventive effects of STB-HO, blocking cell cycle and proliferation in colorectal cancers [24]. Moreover, MFP has been studied for enhancing immune activity and recently reported in many studies. Administrations of such MFP as feed supplements could be induced to in crease immune responses against viral infections [25-27]. As previously reported that the immune-stimulatory ability of MFP can be efficiently suppressed tumors, most studies have investigated MFP for the anti-tumor and the immune-stimulatory effects in cancer stem cells.

Here, we determined the effects of a novel MFP, designated STB-HO, on the teratoma formation of human embryonic stem (hES) cells. First, we investigated whether the effects of STB-HO influenced only undifferentiated cells or differentiated cells as well. We found that STB-HO treatment regulated pro- and anti-apoptotic factors, particularly in early differentiating hES cells; however, the effects of STB-HO were limited in EBs. We next investigated changes in the Bcl-2 protein family. STB-HO treatment of early differentiating hES cells up-regulated pro-apoptotic proteins and pro-apoptotic caspases. Finally, we performed a teratoma formation assay using STB-HO-pretreated differentiating hES cells and showed their preventative effects following subcutaneous transplantation.

## RESULTS

### Changes in pluripotency- and apoptosis-related gene expression upon STB-HO treatment in early differentiating hES cells

We hypothesized that STB-HO could influence the teratoma formation of hES cells by stimulating apoptosis. To identify the suppressive effects of STB-HO, we first cultured hES cells on feeder cells and characterized them based on their alkaline phosphatase activity, which is a well-known, typical characteristic of ES cells. Next, we spontaneously differentiated hES cells, which formed colonies. These spontaneously differentiated hES cell colonies were treated with STB-HO and characterized by quantitative RT-PCR. We assessed changes in gene expression with increasing chemical doses of STB-HO. The gene expression of three pluripotent markers, OCT4, SOX2 and NANOG, demonstrated a tendency toward reduction at all doses (Figure S1A). In particular, the expression of both the OCT4 and SOX2 genes was significantly reduced at the 10 μg/ml dose. In contrast to the three pluripotent markers, two oncogenes, c-MYC and KLF4, which are well-known as iPS-inducing factors, exhibited no particular trend in terms of gene expression with increasing chemical doses of STB-HO (Figure S1B). The oncogenes showed decreased gene expression at the 10 μg/ml dose and increased gene expression at the 100 μg/ml dose. Based on this, we therefore used the 10 μg/ml dose for the following experiments with differentiating and undifferentiated hES cells.

**Figure 1:**
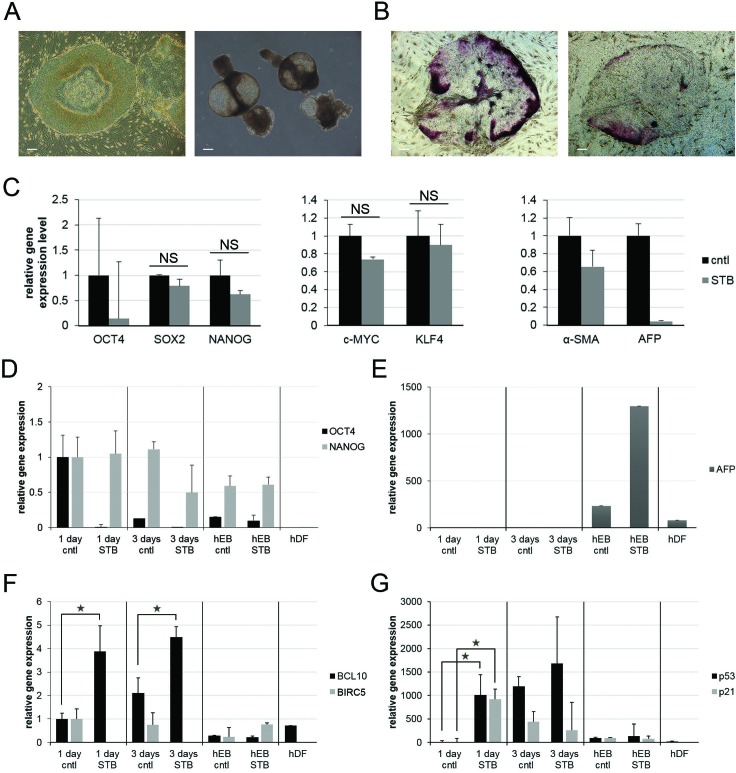
Dynamic changes of pluripotency- and apoptosis-related gene expression patterns upon STB-HO treatment of differentiating hES cells **A.** Undifferentiated hES cells were spontaneously differentiated into following cell types: 1-day and 3-day differentiating hES cells (left) and sphere-shaped hEBs (right). **B.**-**C.** Early spontaneous differentiating hES cells in naïve (B; left) and STB-HO-treated (B; right) colonies were characterized by alkaline phosphatase activity **B.** and by gene expression patterns **C. D.**-**E.** Quantitative real-time RT-PCR analysis of pluripotent marker genes (OCT4 and NANOG), an endodermal differentiation marker gene (AFP) and apoptosis-related genes (BCL10, BIRC5, p53 and p21) was performed on 1-day and 3-day differentiating hES cells and hEBs treated with 10 μg/ml STB-HO. hDFs are shown as controls for fully differentiated cells. Scale bar = 200 μm. *P < 0.05.

To better understand the effects of STB-HO in hES cells, we induced the spontaneous differentiation of hES cells and examined changes in alkaline phosphatase activity and gene expression. As shown in Figure 1A, hES cells can be induced to commence spontaneous differentiation upon withdrawal of FGF2 and can further give rise to sphere-shaped embryoid bodies (EBs) in a suspension culture during the early differentiation stage. In this manner, we differentiated hES cells and individually compared three differentiating hES cell types: 1-day and 3-day differentiating hES cells as well as hEBs. During the early spontaneous differentiation of hES cells, differentiating hES cell colonies showed extended loss of alkaline phosphatase activity after STB-HO treatments (Figure 1B). Nevertheless, the gene expressions of iPS inducing factors excluding OCT4 were not significantly changed (Figure 1C). In detail, the gene expression of OCT4 was rapidly reduced in the 1-day differentiating hES cells, and the gene expression of NANOG was gradually reduced in the hEBs (Figure 1D). With STB-HO, NANOG expression was decreased even in the 3-day differentiating hES cells, and furthermore, the gene expression of alpha-fetoprotein (AFP), an early endodermal lineage marker, was dramatically increased in the hEBs (Figure 1E). These data suggest that STB-HO treatment induced a rapid loss of pluripotency and/or undifferentiated hES cells in early differentiation cultures.

Recently, it has been reported that two anti-apoptotic factors, BIRC5 and BCL10, are preferentially expressed in hES cells [17]. We therefore monitored the gene expression patterns of these two anti-apoptotic factors to determine whether their expression changed during the spontaneous differentiation of hES cells. The gene expression levels of BIRC5 and BCL10 did not differ between vehicle- and STB-HO-treated hEBs, but they were significantly changed in 1-day and 3-day differentiating hES cells (Figure 1F). BCL10 expression was up-regulated by 3.9- and 4.5-fold. Conversely, BIRC5 expression was remarkably decreased and was not detected, respectively. These data led us to presume that STB-HO may stimulate apoptosis in differentiating hES cells by diminishing anti-apoptotic factors, which prevents the activation of apoptosis.

As reported in many studies, the tumor suppressor protein p53 demonstrates differential sensitivity to DNA damage, which leads to apoptosis in hES cells and differentiated cells [29, 30]. However, p53 triggers (activates) mitochondria-mediated apoptosis in hES cells. The up-regulation of p53 and its down-stream target p21 was identified 3 days after the spontaneous differentiation of hES cells (Figure 1G). Interestingly, spontaneous differentiation of hES cells with STB-HO resulted in a rapid change in the gene expression of p53 and p21. In 1-day differentiating hES cells with STB-HO, gene expression levels were approximately 1000-fold higher than the levels without STB-HO. These increases in the gene expression levels of the 1-day differentiating hES cells were significant. Gene expression was not significantly different in other differentiating hES cell types. These findings prompted us to hypothesize that the selective apoptosis of undifferentiated hES cells occurs during early spontaneous differentiation via the activation of p53 and p21, and furthermore, it might be accelerated in the presence of STB-HO.

### No suppressive effects of STB-HO on pluripotency in undifferentiated hES cells

**Figure 2:**
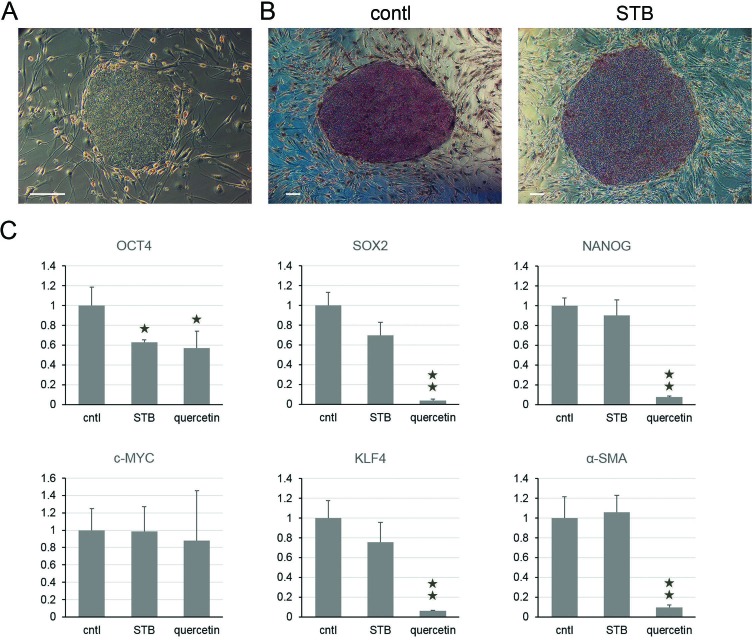
Harmless effects of STB-HO on pluripotency in undifferentiated hES cells **A.** A representative phase-contrast image of hES cells growing on STO feeder cells is shown. **B.** Characterization of hES cells based on alkaline phosphatase activity was performed on naïve (left) and STB-HO-treated (right) undifferentiated hES cell colonies. **C.** Quantitative real-time RT-PCR analysis of pluripotent marker genes (OCT4, SOX2 and NANOG), tumorigenic reprogramming factors (c-MYC and KLF4) and a differentiation marker (α-SMA) was performed in undifferentiated hES cells with treatment of STB-HO and quercetin. Scale bar = 200 μm. *P < 0.05, **P < 0.01.

Now, it is a valid and important question, whether STB-HO does effect on undifferentiated hES cells or not. To answer this issue, we analyzed alkaline phosphatase activity and gene expression patterns with untreated and treated hES cells in the undifferentiated stage (Figure 2A). The hES cells were positively stained for alkaline phosphatase, whereas the feeder cells were negatively stained. The experiments of alkaline phosphatase activity indicated that STB-HO does not influence the undifferentiated state to decrease undifferentiated cell populations in hES cell colonies. By gene expression patterns of iPS-inducing factors, the gene expression of the pluripotent markers SOX2 and NANOG showed no changes by STB-HO treatments, while quercetin treatments dramatically decreased both gene expressions (Figure 2C). In contrast to the differentiating hES cells, two oncogenes c-MYC and KLF4 exhibited no particular trend like two pluripotent markers. In addition, we analyzed further gene expression patterns of apoptosis-related factors, such as p53, p21, BCL10 and BIRC5 (Figure S2). Like iPS-inducing factors, these genes showed not significant changes following STB-HO treatments. After repeated gene expression experiments, we figured out that STB-HO does not influence the undifferentiated stage of hES cells to change pluripotency- and apoptosis-related factors.

### STB-HO regulates commitment to mitochondria-controlled apoptosis in early differentiating hES cells

**Figure 3:**
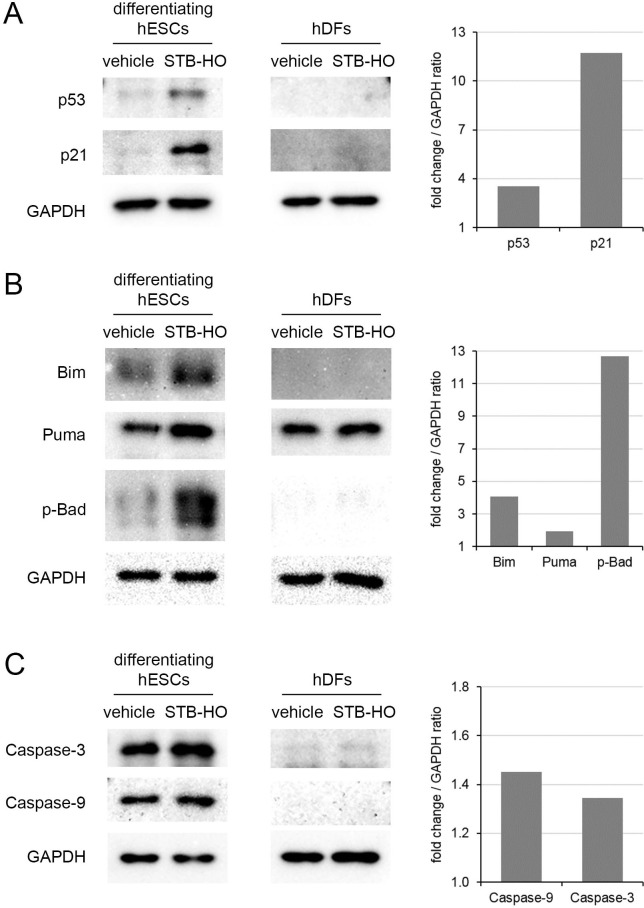
STB-HO regulates commitment to mitochondria-controlled apoptosis in early differentiating hES cells Western blot analyses of **A.** apoptosis-related proteins (p53 and p21), **B.** the Bcl-2 protein family (Bim, Puma, p-Bad) and **C.** apoptosis stimulators (Caspase-3 and Caspase-9) in differentiating hES cells and hDFs were performed and compared between treatment with vehicle and 10 μg/ml STB-HO. Representative western blot bands were quantified using *ImageJ* as well as bar graphs, normalizing to the house-keeping protein GAPDH.

To determine whether the selective apoptosis of remaining undifferentiated hES cells in the differentiating populations can be triggered by STB-HO treatment, we first investigated the molecular mechanisms in vehicle- and STB-HO-treated differentiating hES cells. Similar to the findings described above, expression of the p53 protein and its down-stream target p21 was increased 3.5-fold and 11.7-fold by STB-HO treatment, respectively (Figure 3A). However, the effects of STB-HO on p53 and p21 expression have not been observed in human dermal fibroblasts (hDFs). As much is known about the role of p53 as a direct regulator of mitochondrial apoptotic proteins [31, 32], we next tested and compared the Bcl-2 protein family in vehicle- and STB-HO-treated differentiating hES cells. Interestingly, the Bcl-2 homology 3 (BH3)-only proteins, such as Bim, Puma and Bad, were stimulated by STB-HO and were highly expressed in differentiating hES cells but not in hDFs (Figure 3B). A 4-fold increase in Bim, a 2-fold increase in Puma and a 13-fold increase in phosphorylated-Bad (p-Bad) were sufficient to exceed the apoptotic threshold and commit the differentiating hES cells to apoptosis. This pro-apoptotic switching resulted in activation of the critical effectors Caspase-9 and Caspase-3 (Figure 3C).

Then, we further analyzed the effect of STB-HO on undifferentiated hES cells in protein level. Repeated protein expression experiments indicated that STB-HO-treated undifferentiated hES cells have no expression changes of apoptosis-related factors excluding p53 and Bim, which were decreased after STB-HO treatments (Figure S3). Consequently, the decreased levels of both pro-apoptotic factors does not influence undifferentiated hES cells to exceed the apoptotic threshold. Therefore, we concluded that STB-HO treatment of differentiating hES cells could reduce the remaining undifferentiated hES cells, which can give rise to teratomas upon stem cell transplantation.

### STB-HO pre-treatment prevents teratoma formation after *in vivo* transplantation of differentiating hES cells

**Figure 4:**
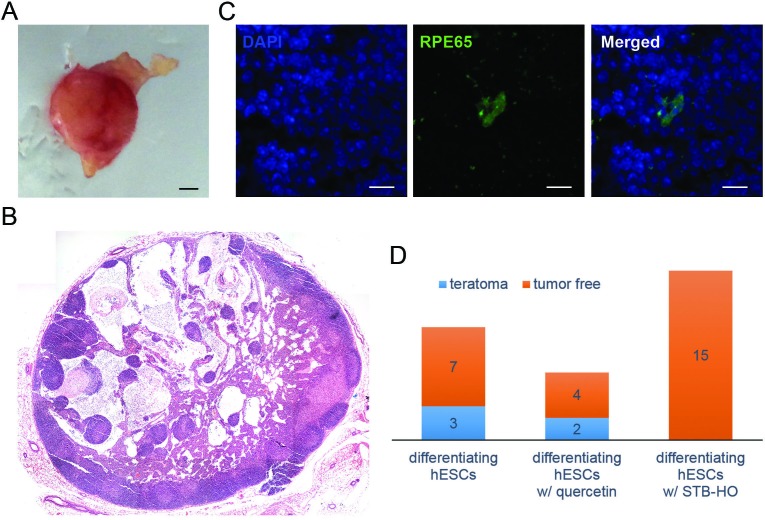
STB-HO pre-treatment prevents teratoma formation after *in vivo* transplantation of differentiating hES cells **A.** After *in vivo* transplantation of differentiating hES cells, tumors were detected at 8 weeks post-transplant. **B.** All tumors were cryosectioned and stained with hematoxylin and eosin. Then, tumors were identified as mature teratoma cases based on whether they were globular shaped and bigger than 5 mm in diameter or as immature teratoma cases if they were smaller and not a typical globular shape. A composite image of a mature teratoma case reveals an entire section of a teratoma that contains elements of three distinct germ layers. **C.** Immunohistochemical images showed human mitochondria-specific marker (HuMi)-positive cells (green) in teratomas developed after xenotransplantations. **D.** The incidence of teratoma formation after *in vivo* transplantation of differentiating hES cells, which were pre-treated with vehicle (*n* = 10), 50 μM quercetin (*n* = 6) or 10 μg/ml STB-HO (*n* = 15), is shown. Scale bar = 1 mm.

As shown above, our data indicated that STB-HO treatment induces the selective apoptosis of remaining undifferentiated hES cells in differentiating populations. Based on these promising results, we performed *in vivo* experiments to determine whether STB-HO pre-treatment of differentiating hES cells could prevent teratoma formation after transplantation. To address this, we used two cell types: differentiating hES cells and hDFs as a negative control. We treated these cells with STB-HO or quercetin for two days before transplantation and subcutaneously injected 1×10^6^ cells into immunosuppressed mice. At two months post-transplant, we assessed teratoma formation in recipient subcutaneous tissues. After sacrificing the mice, we could morphologically detect tumors and isolated them from the implantation site (Figure 4A). To histologically determine the characteristics of the obtained tumors, we stained tissue sections with hematoxylin and eosin and histologically detected typical structures of teratomas that develop from injected hES cells (Figure 4B). To further determine whether teratomas were developed from injected human cells or host tissues, teratoma sections were immunohistochemically stained using human mitochondria-specific antigen HuMi. In teratomas, HuMi-expressing cells mixed with host cells were detected (Figure 4C).

These teratomas could be divided into two categories: 1) mature teratomas, which were globular shaped and bigger than 5 mm in diameter, and 2) immature teratomas, which were smaller and not a typical globular shape [33]. In this manner, the incidence of teratoma formation in the following five groups was determined (Table 1). In the group transplanted only with differentiating hES cells, the incidence as 30%, and all three teratomas were determined to be mature teratoma cases (Figure 4D). In sharp contrast, no teratomas developed from the STB-HO-pretreated differentiating hES cells at the implantation site in 15 recipients. As a control, we performed identical *in vivo* experiments with hDFs and detected no teratomas in 10 recipients for both STB-HO-exposed and STB-HO-unexposed hDF groups. In addition, we compared the inhibitory effects of STB-HO and quercetin on teratoma formation; quercetin has recently been reported to be an effective small molecule for the prevention of teratoma formation from remaining undifferentiated pluripotent cells [17, 34]. However, the quercetin-pretreated differentiating hES cells induced 2 immature teratoma cases out of 6 independent experiments. Taken together, these results strongly support the notion that pre-treatment of differentiating hES cell cultures with STB-HO could inhibit teratoma formation after stem cell transplantation.

**Table 1:** Teratoma formation after subcutaneous injection in mice

Group	Cells injected	Pre-treatment	No. of animals	Teratomas formed
1	Differentiating hESCs		10	30% (3 out of 10)
2	Differentiating hESCs	10 μg/ml STB-HO	15	0% (0 out of 15)
3	Differentiating hESCs	50 μM quercetin	6	33% (2 out of 6)
4	hDFs		7	0% (0 out of 7)
5	hDFs	10 μg/ml STB-HO	3	0% (0 out of 3)

Group 1 comprised all 3 mature teratoma cases and group 3 comprised two immature teratoma cases.

## DISCUSSION

Does STB-HO, a novel MFP, reduce teratoma formation after stem cell transplantation? To answer this question, we first investigated the *in vitro* effects of STB-HO on hES cells. STB-HO regulated pluripotency- and apoptosis-related genes in undifferentiated and also differentiating hES cells. In particular, the expression of the Bcl-2 protein family was markedly changed in early differentiating hES cells after STB-HO treatment. These mitochondrial pro-apoptotic proteins control the up-regulation of the expression of the apoptosis-related proteins p53, p21, and Caspase-3 and -9. Next, we investigated the *in vivo* effects of STB-HO-pretreated hES cells on teratoma formation. The incidence of teratoma formation from differentiating hES cells was 30%, whereas no teratomas developed in 15 recipients that received STB-HO-pretreated hES cells. Thus, our *in vitro* and *in vivo* results might answer the question of whether STB-HO prevents teratomas during stem cell therapy.

In our *in vivo* experiments, the rate of teratoma formation was 30% for early differentiating hES cells (Table 1). This cell population consists primarily of undifferentiated hES cells, which are characterized by alkaline phosphatase activity and the expression of pluripotent marker genes as well as the ability to form teratomas. This teratoma formation of early differentiating hES cells is theoretically expected to occur with a rate of 100%. However, teratoma formation by hES cells according to routine laboratory tests does not demonstrate the theoretical rate, particularly with xenotransplantation. An early study showed that the tumorigenesis of undifferentiated murine ES cells in rats was rare [35]. This *in vivo* study further mentioned that the xenotransplantation of murine ES cells induced no tumor development in intact rats, whereas homologous transplantation induced 95% tumor development. It is therefore likely that the 30% can represent a feasible rate of teratoma formation by xenotransplantation of early differentiating hES cells. Accordingly, we reduced the rate of teratoma formation from 30% (3 out of 10 recipients) to zero (0 out of 15 recipients) by pre-treating with STB-HO.

This study did not fully determine how to induce the selective apoptosis of undifferentiated hES cells. However, we identified key components of mechanisms to regulate apoptosis in hES cells. As it is commonly known, human ES cells, unlike adult stem cells, are vulnerable to DNA damage and consequently are prone to apoptosis. This differential DNA damage sensitivity in hES cells is caused by the apoptotic threshold, which is a balance between cytoplasmic pro- and anti-apoptotic proteins [19, 29]. When pro-apoptotic proteins outnumber anti-apoptotic proteins, hES cells undergo apoptosis. In this apoptotic threshold model, the tumor suppressor p53 plays a crucial role. Departing from its role as a transcription factor, p53 acts to directly interact with pro- and anti-apoptotic proteins in the mitochondria [31]. Consistent with previous findings, STB-HO-mediated activation of p53 induced increases in the expression of its down-stream target p21 and its apoptotic target Puma (Figure 3A-3B), suggesting that the activation of apoptosis dependent on STB-HO was induced by mitochondrial priming.

Conversely, STB-HO treatment regulated BCL10 and BIRC5 (encoding survivin), particularly in the early differentiating hES cells. As mentioned above, hES cells are in close proximity to the apoptotic threshold, which is balanced between pro- and anti-apoptotic proteins, including Puma, Bim, Bad and Bcl-2. These proteins are expressed at higher abundances in hES cells compared to differentiated cells [20, 36]. This apoptotic threshold rapidly disappears during differentiation and then is unable to offer a rapid and direct route to apoptosis [37]. BCL10 and BIRC5 are well-known anti-apoptotic genes and were recently reported to be hES cell-specific expressed proteins [17]. Although it induced a 4-fold up-regulation of BCL10 expression, STB-HO treatment of early differentiating hES cells almost completely inhibited the expression of the BIRC5 gene. These phenomena were observed only in early differentiating hES cells, not in EBs. Taken together, inhibition of BIRC5 by STB-HO implies its ability to rapidly induce the apoptosis of undifferentiated hES cells.

Regarding its effects on differentiation, we administered STB-HO not only to early differentiating hES cells but also to EBs. In general, the endoderm lineage marker AFP is expressed first during the differentiation of hES cells. As expected, AFP expression was detected in EBs. This AFP expression was dramatically elevated after STB-HO treatment, while the apoptosis-related proteins p53, p21, BCL10 and BIRC5 were not significantly changed (Figure C-E). Furthermore, the effects of STB-HO on fully differentiated cells were tested. We treated hDFs with STB-HO and analyzed the expression of these apoptosis-related proteins. Most of them were not expressed and were not up-regulated by STB-HO treatment. Puma was expressed in hDFs but did not change. Our present findings indicate that STB-HO treatment of differentiated cells did not activate apoptosis-related proteins and stimulated the spontaneous differentiation of hES cells. However, the precise mechanism underlying these effects in terms of differentiation and apoptosis remains elusive.

In our cancer study, we investigated the therapeutic effect of STB-HO against cancers, particularly breast cancer [39]. STB-HO treatments exhibited significant suppressive effects on cell growth of MCF-7 breast cancer cell line in a xenotransplantation model, whereas STB-HO did not affect the proliferation and apoptosis of MCF7-cells in cell culture system. Next, the interaction of MCF-7 cells with complex milieu, which consists of tumor microenvironment, was analyzed. Interestingly, STB-HO not only increased the susceptibility of MCF-7 cells to natural killer cells, but also stimulated macrophages and dendritic cells to prevent cancer cells. Thus, these data demonstrate an anti-tumorigenic effect of STB-HO on the suppression of cancer cell growth by regulating of interactions between tumor cells and anti-tumor immune cells.

In conclusion, we focused on the anti-tumorigenic effects of a novel MFP in hES cells. STB-HO could be administered to hES cell cultures and naturally removed when cell colonies were passaged for further differentiation. This short-term STB-HO treatment rapidly induced the selective apoptosis of remaining undifferentiated hES cells in early differentiation cultures. STB-HO blocked an anti-apoptotic gene, BIRC5, and activated p53, p21 and the pro-apoptotic proteins Bim, Puma and p-Bad during early spontaneous differentiation. It can be concluded that STB-HO treatment of early differentiating hES cells can eliminate the remaining undifferentiated hES cells, which are balanced between anti- and pro-apoptotic proteins. Importantly, STB-HO-pretreated differentiating hES cells did not give rise to teratomas with *in vivo* stem cell xenotransplantation. Taken together, our results demonstrate that pre-treatment of differentiating hES cell cultures with STB-HO can prevent teratoma formation after stem cell transplantation.

## MATERIALS AND METHODS

### hES cell culture

hES cell lines (SNUhES3, SNUhES4, SNUhES31; Institute of Reproductive Medicine and Population, Medical Research Center, Seoul National University Hospital, Republic of Korea) were maintained in hES cell medium containing DMEM/F12 supplemented with 20% Knock-out serum replacement, 0.1 mM non-essential amino acids, 0.1 mM β-mercaptoethanol, 50 units/ml penicillin, 50 μg/ml streptomycin and 4 ng/mL bFGF2 on mitomycin C-treated mouse embryonic fibroblast STO (CRL-1503 purchased from ATCC, USA). For spontaneous differentiation, hES cells were cultured with media containing 10% FBS and without bFGF. After 1 week, hES cell colonies were transferred into non-coating dish and further incubated in suspension culture to form hEBs. This study of hES cell lines was approved by the ethics committee SNUIRB (1312/001-006; Seoul National University, Republic of Korea).

### Quantitative real-time RT-PCR

Total RNA was extracted from SNUhES3, SNUhES4 and SNUhES31 cells using TRIzol reagent according to the manufacturer's instructions. The cDNA synthesis and real-time RT-PCR were performed as described previously [38]. Each gene expression was normalized by GAPDH as housekeeping control and relative gene expression levels were calculated using 2^—ΔΔCt^ method. Apoptosis-related primers were used as following: BCL10 (5-TCCTCTCCTTCTTCCCCATT-3, 5-GGCGTCCTTCTTCACTTCAG-3); BIRC5 (5-GGACCACCGCATCTCTACAT-3, 5-GCACTTTCTTCGCAGTTTCC-3); p53 (5-GGCCCACTTCACCGTACTAA-3, 5-GTGGTTTCAAGGCCAGATGT-3); p21 (5-ATGAAATTCACCCCCTTTCC-3, 5-CCCTAGGCTGTGCTCACTTC-3).

### Immunoblotting analysis

Extraction of whole-cell protein lysates and western blotting were performed as described previously [38]. Primary antibodies were used as following; mouse monoclonal p53 (Cell Signaling Technology), rabbit monoclonal Bim, pBad, Caspase-3 (Cell Signaling Technology), rabbit polyclonal p21 (Abcam), Puma, Caspase-9 (Cell Signaling Technology).

### Teratoma formation and cryosectioning

To transplant a mixed population of undifferentiated and differentiated hES cells, SNUhES3 and SNUhES4 cells were spontaneously differentiated in the presence or absence of 10 μg/ml STB-HO for 2 days. 1 × 10^6^ cells were harvested and injected subcutaneously into BALB/cSlc-nu mice (Japan SLC, Inc., Japan). Twelve weeks after injection, xenograft masses were isolated from the skin tissue and fixed for 24 hours in PBS with 4 % paraformaldehyde (Sigma-Aldrich, Germany). Following fixation, tissues were kept in PBS with 16 % glucose (Applichem, Germany), embedded in TissueTek O.C.T. (Sakura Finetek, Heppenheim, Germany) and frozen at −80°C. 10 μm tumor sections were stained with hematoxylin and eosin.

### Statistical analysis

All of the experiments were conducted at least in triplicate (*n* = 3), and the results are expressed as the mean ± SD. Statistical analyses were conducted *via* Student's *t*-test. A value of *P* < 0.05 was considered significant (*P < 0.05; **P < 0.01).

## SUPPLEMENTARY MATERIAL FIGURES


