# Supplementary material for: STB-HO, a novel mica fine particle, inhibits the teratoma-forming ability of human embryonic stem cells after in vivo transplantation
Source: Oncotarget. 2015 Dec 4;7(3):2684–95. doi: 10.18632/oncotarget.6472 (PMC4823064; doi:10.18632/oncotarget.6472)
Supplement: Supplementary file 1 [file oncotarget-07-2684-s001.pdf]

STB-HO, a novel mica fine particle, inhibits the teratoma-forming ability of human embryonic stem cells after *in vivo* transplantation

Supplementary Material

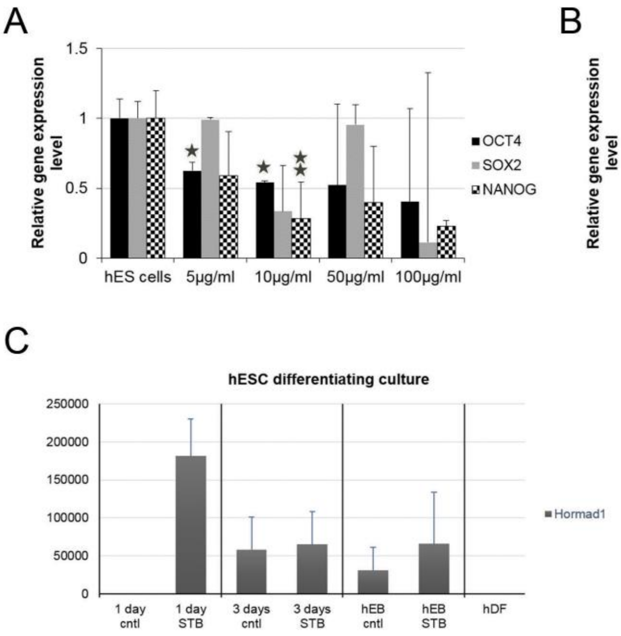

Supplementary Figure S1. Changes of gene expression patterns in early spontaneous differentiating hES cells following STB-HO treatment.

Quantitative real-time RT-PCR analysis of pluripotent marker genes (OCT4, SOX2 and NANOG; A), tumorigenic reprogramming factors (c-MYC and KLF4; B) and an immunogenicity-specific marker (Hormad1; C) was performed in early spontaneous differentiating hES cells with different treatment doses of STB-HO: 5, 10, 50 and 100 µg/ml. $^{*}P < 0.05$, $^{**}P < 0.01$

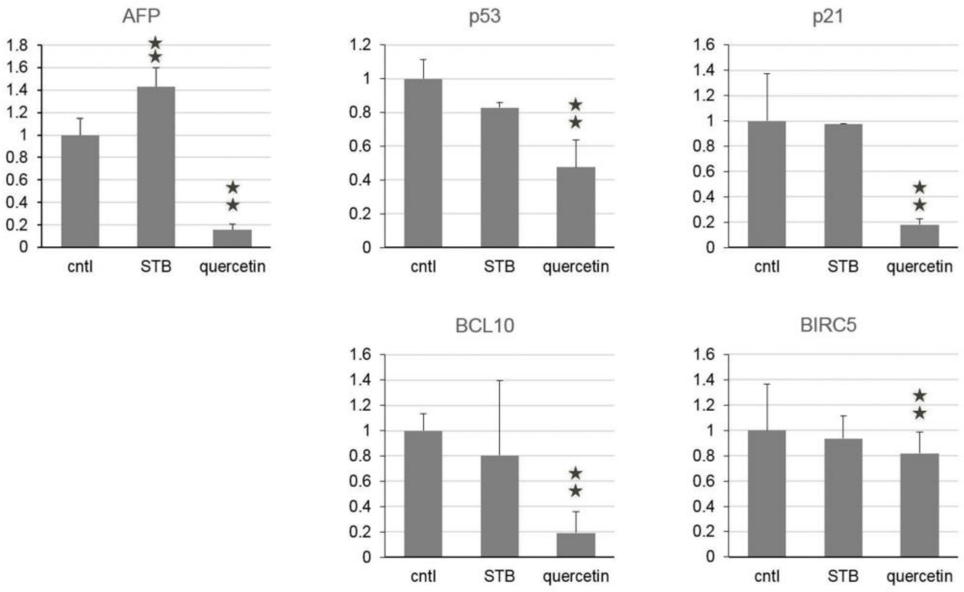

Supplementary Figure S2. Changes of gene expression patterns in undifferentiated hES cells following STB-HO treatments.

Quantitative real-time RT-PCR analysis of an endodermal differentiation marker gene (AFP) and apoptosis-related genes (BCL10, BIRC5, p53 and p21) showed gene expression levels in undifferentiated hES cells with treatment of STB-HO and quercetin. *P < 0.05, **P < 0.01.

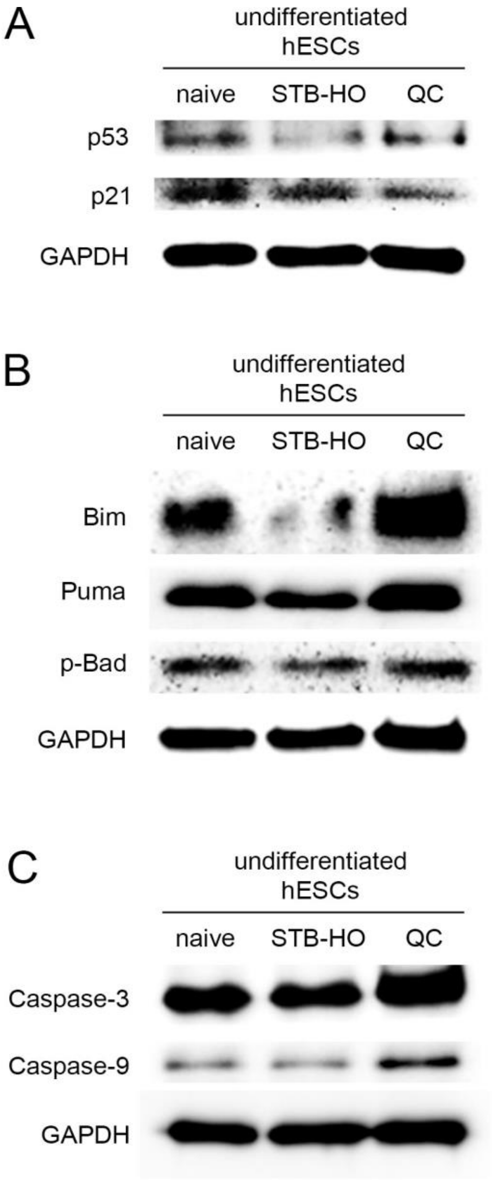

Supplementary Figure 3. Diminutive effect of STB-HO on commitment to mitochondria-controlled apoptosis in undifferentiated hES cells.

Western blot analyses of (A) apoptosis-related proteins (p53 and p21), (B) the Bcl-2 protein family (Bim, Puma, p-Bad) and (C) apoptosis stimulators (Caspase-3 and Caspase-9) in undifferentiated hES cells were performed and compared between naïve, STB-HO-treated and quercetin (QC)-treated cell populations.
